# On the Relations of the Physician to the Sick, to the Public, and to His Colleagues

**Published:** 1847-04-01

**Authors:** 


ON THE RELATIONS OF THE PHYSICIAN TO THE SICK, TO THE PUBLIC, AND TO HIS COLLEAGUES. By the late *C. W. Hufeland,* M.D., &c. 12mo, pp. 37. Oxford, 1847.

THE perusal of this little work will do good to every member of our profession. It is replete with pure and elevated sentiments. How well did the venerable author appreciate the true dignity of Medicine:

"The healing art, therefore, is something sublime and really divine; for its duties coincide with the first and most sacred laws of Religion and philanthropy, and require resignation and an elevation of mind far above worldly desires. None but a really moral man can be a Physician in the true sense of the word, and it is such a one only that can find satisfaction in his vocation; for he alone is conscious of a higher end of existence, which exalts him above earthly considerations and the joys and troubles of life. To improve his mind, to sacrifice his person for the public good and a better world, and to disseminate good around him as much as lies in his power—is what he aims at; and where can he attain that end better than in a profession which gives him daily opportunities, yea, compels him to perform philanthropic acts, acts that are incompatible with selfishness? His professional duties therefore will always beautifully harmonize with his convictions and principles, and, so to say, flow from them. What he ought to do, he does with pleasure; and the consequence will be the highest happiness of man, a consonance of external and internal life. Woe to the Physician, who makes money, or the honour of men, the end of his efforts! He will be in continual contradiction with himself and his duties; he will find his hopes disappointed and his efforts unproductive; he will curse a vocation which does not reward—because he knows not true reward." P. 2.

May such sentiments find a response in the breasts of all!

---

An Inquiry into the Action of Mercury on the Living Body. By *Joseph Swan*. Third Edition, stitched, pp. 34. London, 1847.

